# IGTG&R: An Intent Analysis-Guided Unit Test Generation and Refinement Framework

**DOI:** 10.3390/e28010074

**Published:** 2026-01-09

**Authors:** Xiaojian Liu, Yangyang Zhang

**Affiliations:** 1 College of Computer Science, Beijing University of Technology, Beijing 100124, China; liuxj@bjut.edu.cn; 2 China Electronics Standardization Institute, Beijing 100007, China

**Keywords:** unit test generation, intent analysis, large language model, functional defects

## Abstract

Code coverage-guided unit test generation (CGTG) and large language model-based test generation (LLMTG) are two principal approaches for the generation of unit tests. Each of these approaches has its inherent advantages and drawbacks. Tests generated by CGTG have been shown to exhibit high code coverage and high executability. However, they lack the capacity to comprehend code intent, which results in an inability to identify deviations between code implementation and design intent (i.e., functional defects). Conversely, although LLMTG demonstrates an advantage in terms of code intent analysis, it is generally characterized by low executability and necessitates iterative debugging. In order to enhance the ability of unit test generation to identify functional defects, a novel framework has been proposed, entitled the intent analysis-guided unit test generation and refinement (IGTG&R) model. The IGTG&R model consists of a two-stage process for test generation. In the first stage, we introduce coverage path entropy to enhance CGTG to achieve high executability and code coverage of test cases. The second stage refines the test cases using LLMs to identify functional defects. We quantify and verify the interference of incorrect code implementation on intent analysis through conditional entropy. In order to reduce this interference, the focal method body is excluded from the code context information during intent analysis. Using these two-stage process, IGTG&R achieves a more profound comprehension of the intent of the code and the identification of functional defects. The IGTG&R model has been demonstrated to achieve an identification rate of functional defects ranging from 65% to 89%, with an execution success rate of 100% and a code coverage rate of 75.8%. This indicates that IGTG&R is superior to the CGTG and LLMTG approaches in multiple aspects.

## 1. Introduction

Unit testing is widely regarded as the cornerstone of ensuring software quality, with its core objective being to verify whether code implementations comply with design intentions and specifications. However, in reality, design intentions and specifications are often not properly and fully presented, such as in the case of missing documentation. Despite the existence of partial documentation, descriptions of design intentions and specifications are often inadequate in terms of elaboration of each focal method, thus failing to support the design of unit tests. Consequently, the majority of contemporary unit test generation methodologies are predicated on the generation of unit test cases derived from the implemented code, with the objective of attaining diverse forms of code coverage. These approaches are referred to as the code coverage-guided unit test generation (CGTG) approach [1].

Although the CGTG approach can achieve high coverage and high executability, there are inherent limitations due to the fact that test generation and optimization are guided by coverage analysis of the implemented code. Specifically, the CGTG approach takes the implemented code as the sole verification criterion and uses the coverage of the implemented code as the sole evaluation metric for test cases. It is evident that this renders it impossible to ensure that the test logic aligns with the design intent of the focal method. This issue arises due to interference from the implemented code, which prevents the identification of deviations between code functionality, implementation, and design intent (i.e., functional defects). For example, if the actual implementation of the *add(x, y)* function is *x − y* instead of *x + y*, the CGTG approach, which is devoid of intent analysis, will generate tests based on this erroneous code implementation. As a result, the test logic fails to align with the design intent of the add function, and the functional defect remains undetected. Consequently, the CGTG approach is predominantly employed in the context of regression testing.

In instances where design intentions and specifications are absent, a primary challenge for unit test generation approaches is to generate test cases that not only have high coverage and executability but can also identify functional defects, without being affected by the actual code implementation.

In recent years, the large language model-based test generation (LLMTG) approach has enabled test generation through natural language understanding technologies. Although such approaches can conveniently achieve code intent analysis and test case generation, the initial generation of test cases is generally of a low quality, with relatively low coverage and executability, due to the black-box nature of LLMs [2]. Consequently, iterative debugging has become the prevailing technical approach for the LLMTG method. Nevertheless, the question of its stability remains unresolved [3].

In order to address the aforementioned challenges, a novel intent analysis-guided unit test generation and refinement (IGTG&R) framework is proposed. The process of generating unit tests is divided into two distinct stages: the initial generation of tests and the refinement of these tests. In the test generation stage, the initial test cases are generated using the CGTG approach, with the objective of achieving high coverage and high executability of the test cases. In the test refinement stage, with the objective of identifying functional defects, intent analysis and test case optimization are conducted. These are based on the context of the focal method while excluding its method body. The IGTG&R framework has been demonstrated to effectively achieve complementary advantages between the CGTG approaches and the LLMTG approaches. It has been shown to exhibit high test case executability and high code coverage, as well as the ability to identify functional defects.

The remainder of this paper is structured as follows: Section 2 provides a comprehensive overview of the extant research in this field, together with a detailed analysis of the research approach adopted in the present study. Section 3 elaborates on the technical framework of IGTG&R, meticulously delineating the implementation logic and the pivotal technologies employed at each stage. Section 4 provides evidence to demonstrate the effectiveness of IGTG&R in identifying functional defects in an illustrative case study. Section 5 comprises three experiments that are intended to provide a systematic evaluation and analysis of the IGTG&R approach. Section 6 conducts a qualitative discussion and analyzes the threats to validity as well as the future outlook. Section 7 concludes this paper.

## 2. Related Works

Unit test generation approaches automatically generate unit test cases for the focal method (the specific method under test during unit testing). Each test case typically consists of two parts: a test prefix and a test oracle. The test prefix typically comprises a sequence of method call statements or assignment statements, the purpose of which is to drive the focal method into a testable state. The test oracle, meanwhile, is generally constituted by a series of assertion statements that are designed to ascertain whether the behavior of the focal method is in accordance with the expected specifications. The study of test generation approaches focuses on the generation of test prefixes that cover a greater number of testable states and on the generation of test oracles to more accurately detect compliance with expected specifications. At present, the majority of unit test generation approaches can be categorized into two distinct classifications: traditional code coverage-guided test generation approaches and large language model-based test generation approaches.

**Code coverage-guided test generation (CGTG)**: The CGTG approach aims to achieve different types of code coverage by automatically generating unit tests using strategies based on search strategies [4,5], random strategies [6], or constraint strategies [7,8]. For example, Evosuite [9] uses search and genetic algorithms to generate a test suite that maximizes coverage for a given Java class. Randoop [10] employs a feedback-directed random approach, categorizing test cases into pass or fail suites based on execution results, to generate test cases for Java code. Pynguin [11] applies the techniques used by Randoop [10] to Python code, aiming to address the challenges posed by dynamic typing during test generation.

The CGTG approach involves the analysis of code that has already been implemented through various strategies to maximize test case coverage. Although this approach can achieve high coverage and high executability of test cases, it merely utilizes the coverage of the implemented code as the standard for evaluating test cases. This approach is unable to ensure that the test logic remains consistent with the design intent of the focal methods. This may result in the inability to identify functional defects due to interference from the implemented code.

In comparison with the CGTG approach, the unit test logic generated by the IGTG&R approach, notably the test assertions, remains consistent with the design intent of the code. Conversely, it does not necessarily align with the implemented method’s body code, thereby ensuring the identification of functional defects in the focal method.

**Large language model-based test generation (LLMTG)**: The advent of artificial neural network technology has precipitated a surge in research endeavors concerning the development of test generation approaches founded upon deep learning [12,13,14]. These approaches mostly treat unit test generation as a machine translation problem, translating the given focal method into its corresponding test prefix and test oracle. In recent years, LLMTG approaches [2,3,15] have gradually emerged. They fine-tune pretrained models on test generation tasks by fully leveraging the capabilities of large language models to generate more human-like and meaningful test cases, demonstrating the enormous potential of large language models in unit test generation.

However, test generation approaches based on deep learning and LLMs are plagued by two identified significant issues: (1) Due to the black-box nature of deep learning and LLMs, regardless of code generation or test generation, the problem of ‘generating in one minute, debugging for a whole day’ is severe. Because of inadequate verification mechanisms, the initial generated test cases are generally rough and require substantial manual debugging work. It is evident that iterative debugging has become the prevailing technical approach for LLMTG, but its stability cannot yet be guaranteed [3]. (2) The constraint of context length limits the ability of test generation to process all relevant information and generate comprehensive unit tests. Although recent LLMs have shown the capability to handle longer prompts as input, due to economic costs and intermediate loss effects, providing concise and accurate context information to LLMs remains necessary [15].

In comparison with the emerging LLMTG approach, we innovatively propose the exclusion of the implemented method’s body from the LLM prompts. This is conducted to reduce the interference of code implementation on the analysis of the intent of the focal method and the generation of tests. This approach is effective in identifying functional defects. Furthermore, IGTG&R has been demonstrated to attain a higher Execution Success Rate (ESR) and Code Coverage Rate (CCR).

## 3. IGTG&R Framework

IGTG&R is an intent analysis-guided unit test generation and refinement framework. It has been developed to address issues identified in the CGTG approach, including its inability to identify functional defects, and the unstable quality of the LLMTG approach.

**Figure 1:**
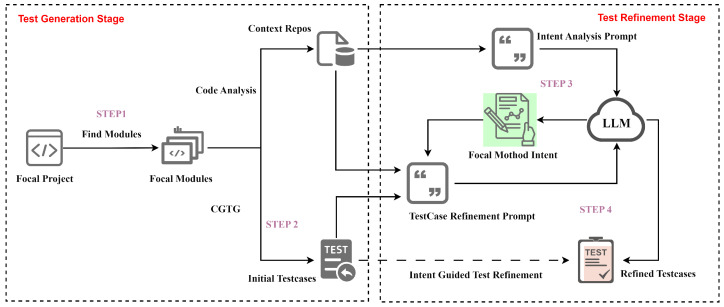
Two-stage architecture of IGTG&R: test generation and refinement.

The IGTG&R approach combines the complementary strengths of two approaches: it employs the high executability and high code coverage of the CGTG approach to offset the instability in generation quality of the LLMTG approach, and it employs the intent analysis capability of LLMTG to compensate for the lack of understanding of design intent in the CGTG approach, thereby enabling the identification of functional defects. The IGTG&R framework consists of a two-stage process for test generation. The first stage employs CGTG to achieve high executability and code coverage of test cases. The second stage refines the test cases using LLMs to identify functional defects. The IGTG&R framework is depicted in Figure 1.

**STEP 1: Identify focal code**: The IGTG&R framework accepts the root directory of the focal project as the initial input, recursively traverses all subdirectories under the specified root directory, and searches for *.py* files that comply with Python package standards (i.e., directories containing the *__init__.py* file or conforming to namespace package standards). IGTG&R then converts their paths into dot-separated module names (e.g., *mypackage.subpackage* corresponds to the *subpackage.py* file under the *mypackage* folder in the root directory of the focal project). The list of focal modules is employed for the purpose of defining the scope of code analysis and test generation.

**STEP 2: Generate initial test cases based on CGTG**: Utilizing random strategies and genetic algorithms, the generation of initial test cases is driven by code coverage analysis of the focal code. Firstly, random strategies are employed to generate a population of test prefixes. These are then checked for validity to ensure that they can execute normally without failing due to syntax or other issues. A fitness function is then defined based on code coverage of the focal code. The genetic algorithm performs selection, crossover, mutation, and other operations on the population of test prefixes, iteratively achieving test cases with high code coverage.

Genetic algorithms are prone to “premature convergence” during iteration, the phenomenon where the code paths covered by individual members of the population become homogeneous, making it difficult to explore new test paths and ultimately limiting the coverage range of the generated test cases. To address this issue, we introduce information entropy theory to quantify population diversity and dynamically adjust genetic algorithm parameters, thereby balancing “diversity maintenance” and “convergence efficiency”. Specifically, we calculate the “coverage path entropy” $H_{\mathrm{pop}}$ of the entire population to measure the diversity of code paths covered by the test prefix population. The calculation formula is as follows:

(1) $$\begin{array}{c} H_{\mathrm{pop}}=-\sum_{p\in P_{\mathrm{all}}}\mathrm{Pr}\left(p\right)\log_{2}\mathrm{Pr}\left(p\right) \end{array}$$

where $P_{\mathrm{all}}$ denotes the set of all code paths covered by individual members of the population, while Pr(p) represents the probability of path *p* appearing in the population (i.e., the proportion of test prefixes that cover path *p* in the population).

We preset an entropy threshold θ (e.g., θ=0.3) to guide parameter adjustment:

- If $H_{\mathrm{pop}}<\theta$, a low entropy value indicates that the paths covered by the population are relatively single (low uncertainty), meaning that the algorithm is at risk of premature convergence. In this case, we increase the crossover/mutation probability to introduce more genetic diversity and encourage the exploration of new test paths.
- If $H_{\mathrm{pop}}>\theta$, a high entropy value indicates sufficient population diversity. We maintain the original crossover/mutation parameters to ensure the population converges toward “high coverage” paths, avoiding excessive randomness that would hinder efficient iteration.

It is noteworthy that during the generation of test cases using CGTG, continuous checks for executability and code coverage are performed on the test cases against the implemented focal code. This approach is intended to guarantee that the generated test cases exhibit both high executability and high code coverage. As was stated previously, CGTG’s limitation can be attributed to its utilization of the implemented code as the exclusive validation criterion. This approach is inadequate in ensuring semantic consistency between code implementation and design intent. Therefore, in *STEP 3* and *STEP 4*, intent analysis and test refinement will be conducted based on the context of the focal code. This will serve to reduce the interference of code implementation on test generation and ensure that the verification logic of the generated tests aligns with the design intent. The outcome of this process is the ability to identify functional defects in the focal code.

**Figure 2:**
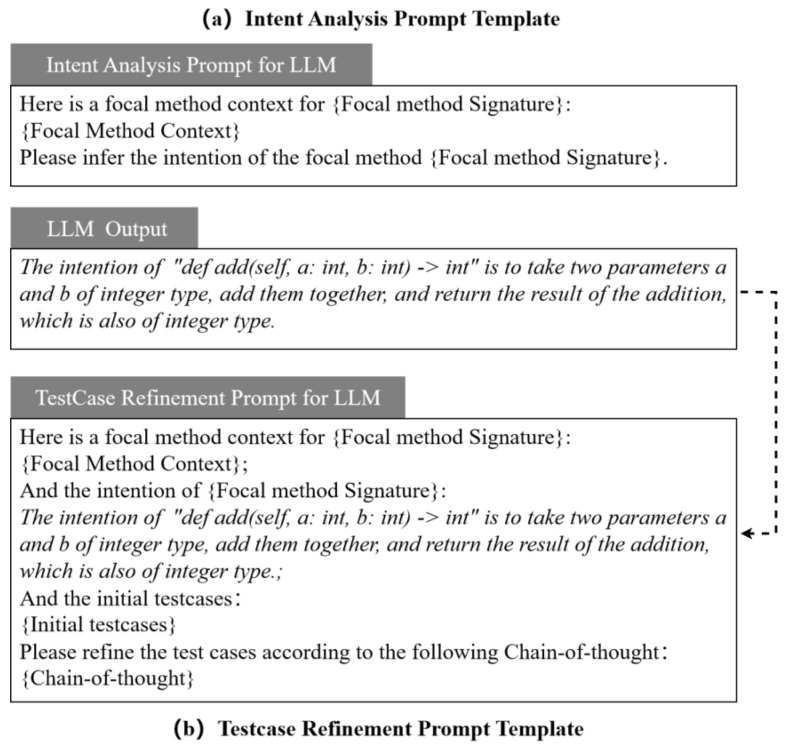
Prompt templates for LLMs.

**STEP 3: Intent analysis based on LLMs**: For each module identified in *STEP 1*, the code will be parsed using the Abstract Syntax Tree (AST) to collect context information for each focal method. This will be used for intent analysis and test refinement. The fields of the context information collected are shown in Table 1. Then, we encapsulate the context information of each focal method as intent analysis prompts and input them into LLMs for intent analysis, as depicted in Figure 2a. It is noteworthy that the context information collected from the focal methods does not include the implementation methods. As demonstrated in Section 5, experiments have shown that the exclusion of the method body has an acceptable impact on the intent analysis. Furthermore, this exclusion can reduce the interference of code implementation on test generation. This, in turn, aids in the identification of functional defects.

**STEP 4: Refine test cases based on intent analysis**: Based on the intent analysis results of the focal method from *STEP 3*, the initial test cases from *STEP 2* will be refined. *STEP 2* has already guaranteed that the test cases have high executability and high code coverage. In *STEP 4*, the emphasis is on refining the test oracle (i.e., assertion statements) to ensure that the validation logic of the test cases aligns with the design intent. Chain-of-Thought (COT) prompting has been demonstrated to be an effective method of enhancing the ability of LLMs to perform engineering tasks [16]. The construction of a test case refinement prompt template is illustrated in Figure 2b. The template for the COT field is outlined below.

(1) *Identify all test assertions in {initial test cases} related to the {focal method’s signature};*
(2) *Analyze whether the identified test assertions related to the {focal method’s signature} conform to the described intent;*
(3) *Only modify those assertions that do not conform to the described intent of the {focal method’s intent} by making them align with the described intent;*
(4) *Re-analyze all assertions related to the {focal method’s intent} to ensure that they can verify the correct implementation of the method’s intent. If not, add assertions to verify the correct implementation of the method’s intent;*
(5) *Output the refined test cases, including comments explaining the refinements.*

## 4. Illustrative Example

We take a simple *Calculator* module as an example to demonstrate the process of generating effective unit tests for identifying functional defects using the IGTG&R approach. In the provided example, the focal method is *def add(self, a:int, b:int)* within the *Calculator* class. The correct implementation should be defined as *return a+b* in order to achieve the functionality of adding two integers. In the provided example, we intentionally write it incorrectly as *return a-b* to serve as the functional defect in this example. The objective of this study is to demonstrate the discrepancies between the tests generated by CGTG and those generated by the IGTG&R approach. This will serve to validate the IGTG&R approach’s functional defect identification capability.

**Figure 3:**
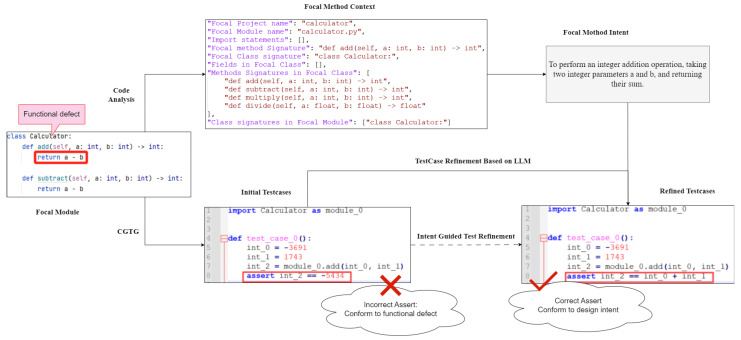
Illustrative example.

As illustrated in Figure 3, IGTG&R initially generates test cases based on CGTG. As previously mentioned, CGTG confines its test generation process to analyzing and inspecting the implemented code itself. This results in the generated test cases having validation logic that aligns with the logic of the implemented code. Consequently, the initial test cases generated essentially verify whether the return value is equal to *a − b*.

Furthermore, the IGTG&R method performs intent analysis and guides the refinement of test cases based on this analysis. Initially, the code analysis is conducted on the focal module, the *Calculator* class, in order to identify the context of the focal method *def add(self, a:int, b:int)*. The intent analysis prompt is then encapsulated using the template shown in Figure 2a, after which it is used by the LLM for intent analysis. As illustrated in Figure 3, the context of the method *def add(self, a:int, b:int)* does not include the implemented method body *return a − b*. This signifies that the LLM is not informed about the implementation details of the method, thereby reducing the interference of code implementation on intent analysis.

Subsequently, the intent analysis result, initial test cases, and COT are to be encapsulated into LLM prompts, as depicted in Figure 2b. The refinement of test cases is then guided by the intent analysis. As illustrated in Figure 3, the refined test cases modify the test assertion in comparison to the initial test cases, thereby achieving validation for the functionality of *return a + b*. In contrast to the initial test cases, which employed the logic of the implemented code as the test verification logic and were unable to identify incorrect implementation (i.e., *return a − b*), the refined test cases employ the design intent of the focal method (i.e., addition functionality) as the test logic, thereby enabling the identification of incorrect implementation (i.e., *return a − b*).

## 5. Evaluation

### 5.1. Experimental Design

The proposed IGTG&R framework integrates the high executability and code coverage advantages of CGTG with the code intent analysis capabilities of LLMTG. It is anticipated that IGTG&R will ultimately generate unit test cases that exhibit high code coverage, high executability, and functional defect identification capabilities. In order to verify the effectiveness of the approach, the experimental design unfolds across three perspectives, namely the accuracy of intent analysis, the functionality of defect detection capability, and the quality of test case metrics, which correspond to the subsequent three research questions (RQs), respectively. Through comparative experiments and quantitative analysis, this study comprehensively addresses the RQs, thereby providing empirical support for IGTG&R’s superiority:

**RQ1: How much impact does the exclusion of the method body have on intent analysis of the focal method?** The purpose of the IGTG&R approach is to generate test cases capable of identifying functional defects; that is to say, it assumes that the implemented method body may have possibilities that do not meet the design intent. Consequently, when conducting intent analysis, the context of the focal method provided to the LLM does not include the implemented method body to avoid interference with intent analysis and test generation. The fundamental question of IGTG&R is whether the intent of the focal method can be accurately analyzed in the absence of the method body. We design a comparative experiment to evaluate the impact of excluding the method body on intent analysis. The experiment will make a comparison between the semantic consistency of the results of intent analysis with and without the method body.

**RQ2: Can the tests generated by IGTG&R effectively detect functional defects?** The practical effectiveness of IGTG&R in identifying functional defects will be evaluated by assessing the test cases generated by IGTG&R. In the experiment, predefined functional defects are artificially injected into the focal project. Subsequently, the test cases generated by the IGTG&R framework are executed, and the results are evaluated in order to ascertain the extent to which these test cases can identify the predefined functional defects.

**RQ3: How do the test cases generated by IGTG&R perform in terms of the Execution Success Rate (ESR) and the Code Coverage Rate (CCR)?** The ESR and CCR are two important metrics for the quality of test cases. Nevertheless, the initial set of test cases generated by LLMTG is frequently regarded as being of substandard quality, along with an inadequate ESR and CCR [10]. This is a key issue that IGTG&R aims to address. The objective of the experiment is to make a comparison and analysis of the improvement effects of the IGTG&R framework in relation to the CGTG and LLMTG approaches using the metrics of the ESR and CCR.

The source code of five open-source Python projects was collected as the experimental dataset. The process of selecting the dataset is as follows: From the PyPI repository, select Python projects with available source code. Exclude projects that rely on C/C++ and other languages to avoid cross-language parsing interference and reduce dependencies on compilation environments, thereby ensuring the normal processing of syntax trees and bytecode. Further, use the ’pipdeptree’ tool to sort out the dependency relationships, exclude projects that rely on local code repositories, and ensure that all dataset dependencies come from public sources. At the same time, consider the project size, covering small- to medium-sized projects. The details of each project are shown in Table 2. The dataset projects cover small- to medium-sized projects, with the code size (LOCs) ranging from 85 to 1715 lines, the number of modules ranging from 2 to 36, and the number of methods ranging from 16 to 278. In the experiment, Pynguin [11] was employed as the baseline method for the CGTG approach, represented by CGTG_Pynguin, and ChatGPT [17], CodeLlama [18], and DeepSeek [19] were employed as the LLMs for the LLMTG approach, represented by LLMTG_ChatGPT, LLMTG_CodeLlama, and LLMTG_DeepSeek, respectively. The IGTG&R methods based on different large models are represented by IGTG&R_ChatGPT, IGTG&R_CodeLlama, and IGTG&R_DeepSeek, respectively.

The IGTG&R framework is implemented as a Python application, leveraging modular design to support its two-stage workflow. Core modules include the following: a code-parsing module based on Python’s built-in ‘ast’ for extracting the focal method context, an initial test generation module integrated with Pynguin for CGTG-based generation, an LLM interaction module leveraging Ollama and OpenAI libraries for intent analysis, and a test refinement module implementing Chain-of-Thought logic. For the genetic algorithm (GA) in initial test generation, key parameters are configured as follows (empirically validated for stable performance): an initial population size of 50, a single-point crossover with a crossover probability of 0.75, a uniform mutation with a probability of 1/L (L denotes the number of statements in the test case), and tournament selection with a tournament size of five. Guided by the preset coverage path entropy threshold θ=0.3, crossover and mutation probabilities are dynamically adjusted. When $H_{\mathrm{pop}}<\theta$ (risk of premature convergence), the crossover probability is increased by 30% to enhance diversity; when $H_{\mathrm{pop}}>\theta$, base parameters are retained to ensure efficient convergence toward high-coverage paths.

The experimental environment for tool deployment and execution is specified as follows: one server with an Intel Xeon Silver 4310 processor and an NVIDIA A100-SXM-80GB GPU, as well as equipped with the NVIDIA Driver 535.104.05, CUDA 12.2, Ubuntu 22.04 LTS, and Python 3.10. Pynguin-0.39.0 is locally deployed, and CodeLlama (tag: codellama:34b, date: August 2023) and DeepSeek (tag: deepseek-coder:33b, date: October 2023) are locally deployed via Ollama-0.13.0. ChatGPT (tag: gpt-3.5-turbo-0613, date: November 2022) is accessed via OpenAI’s Chat Completions API version 1.0.

### 5.2. RQ1: How Much Impact Does the Exclusion of the Method Body Have on Intent Analysis of the Focal Method?

**Figure 4:**
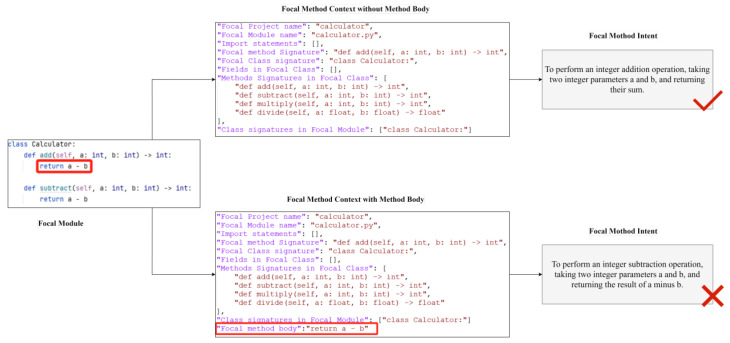
Impact of the incorrect method body on intent analysis.

The IGTG&R framework, in the course of conducting intent analysis, does not include the implemented method body within the context provided to the LLM to avoid any potential interference from the implemented method body with the intent analysis. In order to evaluate the impact of excluding the method body on the intent analysis, a series of experiments was conducted from two perspectives:

(1) *RQ1-1: Can the erroneous method bodies interfere with the results of intent analysis?* We continue to use the incorrect implementation of the *def add(self, a:int, b:int)* method from the illustrative example to compare and analyze the impact of whether the focal method’s body field is included in the context of the focal method on the results of intent analysis. The focal method context and intent analysis prompts are encapsulated in two ways. The only difference between the two is that one includes the focal method’s body field, while the other does not. The two prompts are to be input into ChatGPT, CodeLlama, and DeepSeek for intent analysis. The three large models provide similar results, as illustrated in Figure 4: the prompt without the focal method’s body field correctly identifies the ‘*addition*’ intent, while the prompt with the focal method’s body field incorrectly identifies the ‘*subtraction*’ intent. This finding suggests that erroneous method bodies have the potential to interfere with the results of intent analysis.

To further quantify the degree of interference caused by erroneous method bodies on intent analysis, we introduce conditional entropy H(I|M), which is mathematically defined as the expected value of the entropy of the intent analysis result set *I* under each method body state m∈M:

(2) $$\begin{array}{cc} \; & H\left(I|M\right)=\sum_{m\in M}P\left(M=m\right)\cdot \\ \; & \left[-\sum_{i\in I}P\left(I=i|M=m\right)\log_{2}P\left(I=i|M=m\right)\right] \end{array}$$

where

- The intent analysis result set *I* represents all possible outcomes of intent analysis for the focal method:
  $I_{c}$: Correctly intent analysis.
  $I_{e}$: Incorrectly intent analysis.
  $I_{a}$: Ambiguously intent analysis.
- The method body state set *M* represents all possible states of the method body included in the intent analysis context:
  $M_{c}$: The correct method body.
  $M_{e}$: The crroneous method body.
  $M_{\emptyset }$: No method body.

Ten focal methods were randomly selected from the methods of the five projects listed in Table 2. For each selected focal method, three types of method body states were constructed: $M_{c}$ (original method implementation included in the intent analysis prompt), $M_{e}$ (the method implementation was artificially modified to deviate from its original design intent, e.g., altering an addition function to perform subtraction), and $M_{\emptyset }$ (no method body included in the intent analysis prompt). Three LLMs (gpt-3.5-turbo-0613, codellama:34b, and deepseek-coder:33b) were employed for intent analysis, along with consistent prompt templates to ensure an identical context. Each intent analysis was executed five times to reduce random errors, and outputs were categorized into three intent analysis results ($I_{c}$, $I_{e}$, and $I_{a}$).

The distribution of the intent analysis results is shown in Table 3. $H(I|M_{e})=1.38$ is approximately four times higher than $H(I|M_{c})=0.33$ and approximately two times higher than $H(I|M_{\emptyset })=0.64$, directly validating that erroneous method bodies severely mislead LLM-based intent analysis. In $M_{e}$, over half of the intent analysis results (56.7%) deviate from the original intent ($I_{e}$), and nearly 30% are ambiguous ($I_{a}$). This confirms that retaining erroneous implementations have a significant impact on the intent analysis of the focal method.

The IGTG&R method is centered on the generation of tests for functional defects. The exclusion of the implemented method body from the context information of the focal method has been demonstrated to prevent incorrect implementations from affecting the intent analysis of the focal method. Moreover, it will be demonstrated that this method can enhance the identification of functional defects in RQ2.

**Figure 5:**
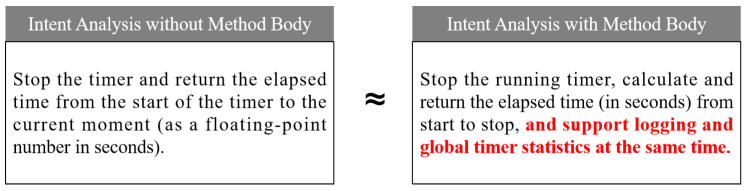
An example of overlooking non-core intent logic due to the exclusion of the method body.

(2) *RQ1-2: Does the exclusion of the method body result in bias in the intent analysis?* The intent analysis prompts for all methods in Table 2 were encapsulated in two ways: the inclusion of the focal method body field and its exclusion. And no deliberate injection of functional defects was conducted in the method body. The two types of prompts were input into ChatGPT, CodeLlama, and DeepSeek for intent analysis. The results were then compared to assess semantic consistency. Figure 5 illustrates the intent analysis result of the *def stop(self) ->float* method in the *timer.py* files of the *codetiming* project with and without the method body. Ignoring the details of functional implementation descriptions, both approaches correctly captured the core intent of the focal methods and maintained semantic consistency. Therefore, it can be concluded that the impact of excluding the method body on the intent analysis is within an acceptable range.

**Summary for RQ1**: The exclusion of the method body does not have a significant impact on the intent analysis of the focal method. Moreover, this exclusion has the effect of reducing the interference of code implementation on intent analysis and test generation, thus enhancing the identification of functional defects.

### 5.3. RQ2: Can the Tests Generated by IGTG&R Effectively Detect Functional Defects?

In order to evaluate the practical effectiveness of the IGTG&R approach in detecting functional defects, the focal project was artificially injected with predefined functional defects, and test cases were generated using both IGTG&R and CGTG. A comparison of the results was conducted to evaluate the efficacy of the IGTG&R approach in identifying the injected functional defects. For the focal projects listed in Table 2, the focal methods are selected at random, and functional defects are injected through code mutation. The mutation types include the following:

- Arithmetic operator replacement: e.g., + → − or ∗ → /.
- Logical operator replacement: e.g., and → or or == → !=.
- Statement deletion: Remove a line of code (such as a return statement).
- Constant value replacement: e.g., True → False or 1 → 0.
- Variable name replacement.

The injection and identification of functional defects are shown in Table 4. The experimental results confirmed the inherent limitations of the CGTG approach in identifying functional defects. The CGTG method generates test cases based on code implementations with injected functional defects, leading to verification logic that aligns with the defective code’s implementation. Consequently, it fails to identify deviations between the code’s implementation and the design intent. In contrast, IGTG&R exhibits a superior capacity to identify functional defects, with an identification rate ranging from 65% to 89%.

A detailed analysis of the functional defects that the IGTG&R failed to identify was conducted. Generally, this phenomenon occurred in instances where code mutations resulted in internal logic errors, without compromising the effective implementation of the intended functionality of the focal method. In other words, the functional implementation of the code was consistent with the intent analysis from the perspectives of both input and output. That is, the code mutations did not result in changes to the input and output of the focal method; thus they could not be identified by the IGTG&R approach. This finding suggests that while the exclusion of the method body does not affect IGTG&R’s capacity to correctly capture the core intent of the focal method, it indeed overlooks certain code details that do not impact the core functional intent.

Figure 5 shows the intent analysis result of the *def stop(self) ->float* method in the *timer.py* files of the *codetiming* project with and without the method body. The method body of *def stop(self) ->float* not only implements the timing function but also the additional logging function. Since the logging function is not the typical core function, the LLM is unable to predict this design intent without the method body. In the experiment, the code mutation occurred precisely in the code corresponding to the logging function. Consequently, the IGTG&R method fails to focus on the logging function, based on the intent analysis result. Accordingly, it is incapable of refining the test cases and consequently unable to identify the defect.

**Summary for RQ2**: In comparison to the CGTG method, the IGTG&R framework has been demonstrated to exhibit better capability in identifying functional defects. Nevertheless, IGTG&R does have certain limitations. Due to the exclusion of the method body during intent analysis, IGTG&R may overlook certain code details that do not impact its core functionality. In the event of functional defects arising in these components of the code, the test cases generated by IGTG&R may be ineffective in identifying these defects.

### 5.4. RQ3: How Do the Test Cases Generated by the IGTG&R Method Perform in Terms of the Execution Success Rate (ESR) and the Code Coverage Rate (CCR)?

**Figure 6:**
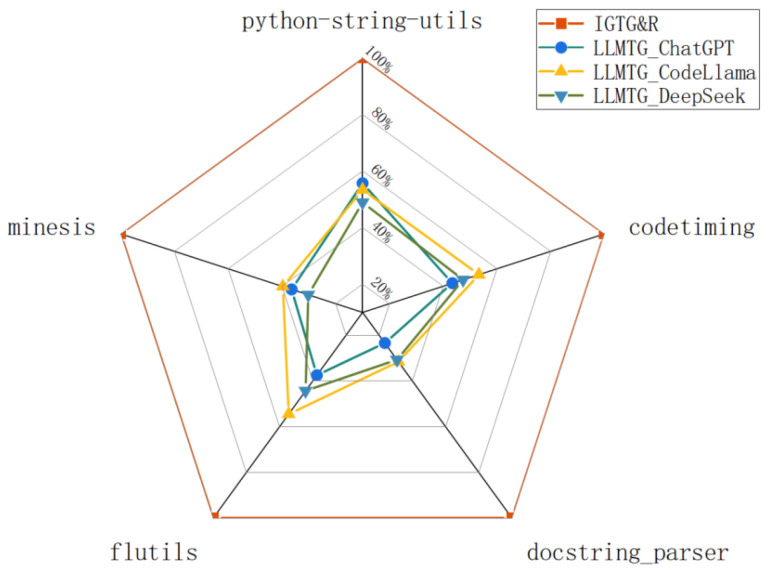
Comparative analysis of the Execution Success Rate (ESR).

**Figure 7:**
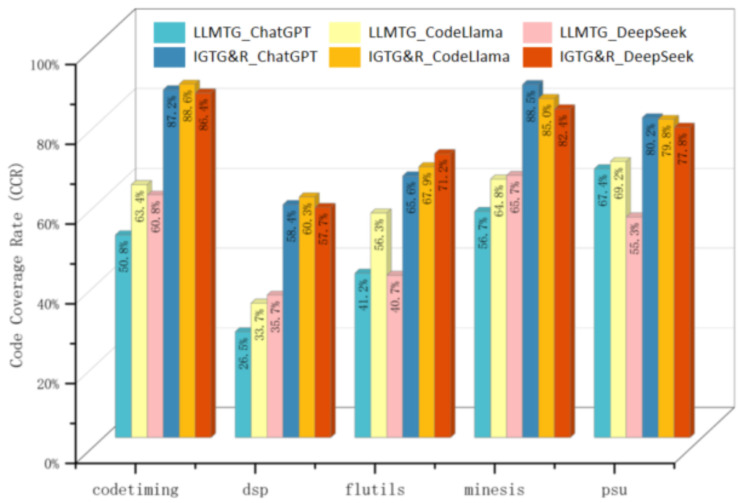
Comparative analysis of the Code Coverage Rate (CCR).

The improvement effects of the IGTG&R method on the CGTG and LLMTG approaches are compared and analyzed from two perspectives: the Execution Success Rate (ESR) and the Code Coverage Rate (CCR). In this experiment, the ESR denotes the proportion of generated test files that can run without manual debugging (i.e., no ’ERROR’ causes the test execution to stop), while the CCR denotes the branch coverage of the generated test cases. CGTG employs Pynguin [11] as the baseline method to generate tests and is represented by CGTG_Pynguin, while LLMTG approaches generate tests based on ChatGPT [17], CodeLlama [18], and DeepSeek [19] and is represented by LLMTG_ChatGPT, LLMTG_CodeLlama, and LLMTG_DeepSeek, respectively. A range of IGTG&R variants that were based on ChatGPT, CodeLlama, and DeepSeek was implemented for the purpose of a comparative ablation study. These variants are represented by IGTG&R_ChatGPT, IGTG&R_CodeLlama, and IGTG&R_DeepSeek, respectively. The experimental results are shown in Table 5, from which the following analysis can be derived:

(1) *Execution Success Rate:* It was observed that both CGTG and the IGTG&R miraculously achieved a 100% ESR, which is attributed to the utilization of Pynguin [11] as the benchmark method for CGTG. Pynguin [11] repeatedly executes test variants during the generation process and records the output results, discarding those tests which are non-executable. This approach guarantees that the generated test files are executable. In stark contrast, the LLMTG method generally exhibits a low ESR for the generated tests (an average of 42%), as illustrated in Figure 6, thereby validating the phenomenon of `generating in one minute, debugging for a whole day’.

(2) *Code Coverage Rate:* The CGTG method generates test cases guided by code coverage, resulting in tests with a high CCR (an average of 72.3%). This impressive performance has been inherited by the IGTG&R framework because IGTG&R uses CGTG to generate initial test cases, ensuring the coverage of the test prefix for the focal code. In addition, the refinement of the LLM during the test refinement phase has resulted in an improvement ranging from approximately 3% to 75.8%. This enhancement can be attributed to the addition of new test prefixes and assertions. In stark contrast, the LLMTG method produces test cases with a generally low CCR (an average of 52.5%), which is largely attributed to a lower ESR. The comparative analysis of the Code Coverage Rate (CCR) between LLMTG and IGTG&R is depicted in Figure 7. It is evident that the test cases generated by LLMTG exhibit excellent functional validation logic. However, the low ESR of the initial test cases results in suboptimal CCR performance. Iterative debugging may result in improvements in the ESR, which would consequently lead to corresponding increases in the CCR; however, this is beyond the scope of this paper.

We conducted statistical tests on the CCR (the ESR has no variance for IGTG&R and CGTG, so no test is needed). A Kruskal–Wallis H test was performed on the CCR across seven approaches, as shown in Table 5, showing a significant overall difference (H = 92.47, *p* < 0.001). Post hoc Dunn tests indicate that each IGTG&R variant’s CCR is significantly higher than that of the corresponding LLMTG variant (*p* < 0.05 for all pairs), while there is no significant difference between IGTG&R variants and CGTG (*p* > 0.05). The 95% confidence intervals (t-distribution) for the average CCR are as follows: CGTG [67.5%, 77.1%], LLMTG (average) [48.2%, 56.8%], and IGTG&R (average) [72.3%, 79.3%]. These results statistically confirm that IGTG&R inherits CGTG’s high-coverage advantage and significantly outperforms LLMTG in terms of the CCR.

**Summary for RQ3**: The CGTG approach has the advantage of a high ESR and CCR, but its functional verification logic in test cases is insufficient. The LLMTG method has excellent intent analysis capability and functional verification logic, but the ESR and CCR of its test cases are generally low. The IGTG&R framework has been demonstrated to efficaciously realize the complementary advantages of both approaches: the CGTG method is utilized to achieve an elevated ESR and CCR of the test cases, while LLMs are employed to refine the test cases and enable the identification of functional defects.

## 6. Discussion and Future Outlook

Existing unit test generation approaches are predominantly divided into two paradigms: CGTG and LLMTG. CGTG approaches are good at producing test cases with high executability and code coverage but fail to align with design intent, limiting their ability to detect functional defects. LLMTG approaches leverage natural language understanding to capture code intent but suffer from low executability and unstable quality due to the black-box nature of LLMs. The proposed IGTG&R framework combines the advantages of CGTG and LLMTG while also compensating for their respective shortcomings. In comparison with the CGTG approach, the unit test logic generated by the IGTG&R approach, notably the test assertions, remains consistent with the design intent of the code. Conversely, it does not necessarily align with the implemented method’s body code, thereby ensuring the identification of functional defects in the focal method. In comparison with the emerging LLMTG approach, we innovatively propose the exclusion of the implemented method body from the LLM prompts. This is conducted to reduce the interference of code implementation on the analysis of the intent of the focal method and the generation of tests. The illustrative example confirmed that IGTG&R is effective in identifying functional defects. The statistical data from the evaluation experiment demonstrated that the identification rate of functional defects ranged from 65% to 89%. Furthermore, IGTG&R has been demonstrated to attain a higher Execution Success Rate and Code Coverage Rate, with an Execution Success Rate of 100% and a Code Coverage Rate of 75.8%. This impressive performance has been inherited from CGTG and further enhanced by the intent analysis capability of LLMTG.

Three types of threats to validity can be identified:

*Internal validity:* In recent years, various types of LLMs have been emerging one after another, and their capabilities have also been continuously enhanced. More recent LLMs have demonstrated significant advancements in code understanding, intent prediction accuracy, and reductions in hallucinations, which could potentially improve intent analysis and test refinement in IGTG&R. However, the core architecture of IGTG&R—leveraging CGTG for high executability/coverage and LLMs for intent-driven refinement—remains robust regardless of model generation. The framework’s critical design choice of excluding method bodies to mitigate implementation interference is equally relevant for newer models, as erroneous code still poses bias risks. While updated LLMs may enhance defect detection precision, they would not alter the framework’s fundamental effectiveness, ensuring the study’s core conclusions remain generalizable.

*External validity:* Another threat lies in the representativeness of the experimental dataset. The selected projects vary intentionally in code size (85–1715 LOCs), the number of modules (2–36), the number of methods (16–278), and application domains, and they are all real Python projects. This diversity aligns with similar unit test generation studies, which often use 4–6 projects for evaluation. While expanding to more projects or additional domains could further strengthen generalizability, the consistent performance of IGTG&R across the diverse sample demonstrates its applicability to common Python project scenarios, mitigating risks of dataset limitation.

*Construct validity*: The construction of ‘functional defects’ is achieved through the process of code mutations (e.g., arithmetic operator replacement or statement deletion). However, this may not fully capture all real-world functional defects (e.g., logical flaws in complex business logic that do not manifest in input–output mismatches). As observed in RQ2, IGTG&R fails to detect defects in non-core logic because the design intent analysis is focused on core functionality, overlooking auxiliary features. Presently, a satisfactory resolution remains elusive. Introducing code annotation or supplementing function descriptions might be a potential solution.

In the future, the IGTG&R framework can be integrated into the broader paradigm of continuous, interactive learning in software engineering, making it a foundational component of an evolving software ecosystem. This also aligns with the latest advancements in AI-driven software engineering [20]. A key direction is the incorporation of Reinforcement Learning with Human Feedback (RLHF), which draws on foundational work in instruction following [21] and crowd-sourced feedback alignment for code generation [22]. This formalizes developers’ assessments of generated tests into structured reward signals, creating a closed-loop system that iteratively fine-tunes the LLM refinement stage to better align with expert priorities. For instance, feedback on whether a test effectively uncovers critical functional defects or avoids redundant assertions will update the reward function. This guides the LLM to generate more practically useful test oracles over time and ultimately optimizes code generation. Beyond RLHF integration, IGTG&R will leverage context-based Retrieval-Augmented Generation (RAG) to enrich intent analysis and test refinement. By retrieving relevant historical test cases, code documentation, and developer feedback from the project’s evolving ecosystem, the framework will enhance the contextual awareness of both the CGTG initial generation and LLM refinement stages. This synergy of RLHF and RAG will enable IGTG&R to adapt to project-specific coding standards, debugging workflows, and domain-specific defect patterns, transforming it into a dynamic, collaborative tool that grows with the software lifecycle. Additionally, extending this interactive learning paradigm to cross-language support and integration with CI/CD pipelines will further solidify IGTG&R’s role as a foundational technology for AI-assisted software testing, bridging the gap between automated test generation and real-world developer needs.

## 7. Conclusions

The present paper proposes an intent analysis-guided unit test generation and refinement (IGTG&R) framework. It effectively integrates the advantages inherent in code coverage-guided unit test generation (CGTG) approaches and large language model-based test generation (LLMTG) approaches. It has been demonstrated that IGTG&R-generated tests exhibit high executability and code coverage and possess the capacity to identify deviations between code implementation and design intent (i.e., functional defects).

Further research may include the following: Firstly, IGTG&R is solely applicable to programs written in the Python language. Its applicability within the context of other programming languages necessitates further exploration and verification. Secondly, as indicated by the evaluation in RQ2, the exclusion of the method body during intent analysis will inhibit the identification of specific functional defects. Determining how to strike a balance between the technical considerations associated with these two aspects will constitute the focus of subsequent work. Additionally, the capabilities of LLMs are undergoing rapid evolution. If the issue of low executability can be resolved, it will engender a comprehensive enhancement in performance, and we will remain attentive to this development. Additionally, IGTG&R has the potential to integrate into the broader framework of continuous interactive learning within software engineering and to become a fundamental component of the evolving software ecosystem.

## Figures and Tables

**Table 1:** Focal method context for intent analysis.

Field	Field Description
Focal Project Name	Name of the project containing the focal method
Focal Module Name	Name of the module containing the focal method
Import Statements	List of import statements for the module containing the focal method
Focal Class Signature	The signature of the class containing the focal method
Fields in Focal Class	List of member variables in the class containing the focal method
Focal Method Signature	The signature of the focal method
Methods Signatures in Focal Class	List of signatures of other methods in the class containing the focal method
Class Signatures in Focal Module	List of signatures of other classes in the module containing the focal method

**Table 2:** Details of focal projects.

Project Name	Version	LOCs	Modules	Methods
codetiming	1.2.0	85	2	16
docstring_parser	0.7.1	608	3	31
flutils	0.6	1715	3	32
minesis	4.0.0	1663	36	278
python-string-utils	1.0.0	476	3	22

**Table 3:** The distribution of the intent analysis results.

Method Body State	$I_{c}$	$I_{e}$	$I_{a}$	H(I|M)
$M_{c}$	94.5	0.5	5.0	0.33
$M_{e}$	13.8	56.7	29.5	1.38
$M_{\emptyset }$	88.2	4	7.8	0.64

**Table 4:** Injection and identification of functional defects.

Project Name	Injected Methods	Identified Methods	
		CGTG	IGTG&R
codetiming	9	0 (0%)	6 (66.7%)
docstring_parser	16	0 (0%)	12 (75%)
flutils	18	0 (0%)	15 (83.3%)
minesis	127	0 (0%)	89 (70%)
python-string-utils	13	0 (0%)	8 (69.2%)

**Table 5:** Statistical results of the Execution Success Rate (ESR) and the Code Coverage Rate (CCR).

Approach		Metric	Project				
			Codetiming	Docstring_Parser	Flutils	Minesis	Python-String-Utils
CGTG	CGTG_Pynguin	ESR	100.0%	100.0%	100.0%	100.0%	100.0%
		CCR	86.4%	55.3%	63.7%	82.4%	73.8%
LLMTG	LLMTG_ChatGPT	ESR	43.4%	23.4%	37.5%	36.4%	55.7%
		CCR	50.8%	26.5%	41.2%	56.7%	67.4%
	LLMTG_CodeLlama	ESR	53.3%	31.6%	54.5%	39.7%	53.4%
		CCR	63.4%	33.7%	56.3%	64.8%	69.2%
	LLMTG_DeepSeek	ESR	47.4%	30.7%	44.3%	30.2%	48.9%
		CCR	60.8%	35.7%	40.7%	65.7%	55.3%
IGTG&R	IGTG&R_ChatGPT	ESR	100.0%	100.0%	100.0%	100.0%	100.0%
		CCR	87.2%	58.4%	65.6%	88.5%	80.2%
	IGTG&R_CodeLlama	ESR	100.0%	100.0%	100.0%	100.0%	100.0%
		CCR	88.6%	60.3%	67.9%	85.0%	79.8%
	IGTG&R_DeepSeek	ESR	100.0%	100.0%	100.0%	100.0%	100.0%
		CCR	86.4%	57.7%	71.2%	82.4%	77.8%

## Data Availability

The data that support the findings of this study are available from the corresponding author upon reasonable request.

## References

[1] Alonso J.C., Segura S., Ruiz-Cortés A. AGORA: Automated generation of test oracles for REST APIs. Proceedings of the 32nd ACM SIGSOFT International Symposium on Software Testing and Analysis.

[2] Yuan Z., Lou Y., Liu M., Ding S., Wang K., Chen Y., Peng X. (2023). No more manual tests? evaluating and improving ChatGPT for unit test generation. arXiv.

[3] Alshahwan N., Chheda J., Finogenova A., Gokkaya B., Harman M., Harper I., Marginean A., Sengupta S., Wang E. Automated unit test improvement using large language models at meta. Proceedings of the 32nd ACM International Conference on the Foundations of Software Engineering.

[4] McMinn P. (2004). Search-based Software Test Data Generation: A Survey. J. Softw. Testing, Verif. Reliab..

[5] Fraser G., Arcuri A. (2011). EvoSuite: Automatic test suite generation for object-oriented software. Proceedings of the SIGSOFT/FSE’11 19th ACM SIGSOFT Symposium on the Foundations of Software Engineering (FSE-19) and ESEC’11: 13th European Software Engineering Conference (ESEC-13).

[6] Duran O.W.J., Ntafos S.C. (1984). An Evaluation of Random Testing. IEEE Trans. Softw. Eng..

[7] Csallner C., Tillmann N., Smaragdakis Y. (2008). DySy: Dynamic symbolic execution for invariant inference. Proceedings of the 30th International Conference on Software Engineering (ICSE 2008).

[8] Ernst M.D., Perkins J.H., Guo P.J., McCamant S., Pacheco C., Tschantz M.S., Xiao C. (2007). The Daikon system for dynamic detection of likely invariants. Sci. Comput. Program..

[9] Gao S., Wen X., Gao C., Wang W., Zhang H., Lyu M.R. (2023). What Makes Good In-Context Demonstrations for Code Intelligence Tasks with LLMs?. Proceedings of the 38th IEEE/ACM International Conference on Automated Software Engineering, ASE 2023.

[10] Pacheco C., Lahiri S.K., Ernst M.D., Ball T. (2007). Feedback-Directed Random Test Generation. Proceedings of the International Conference on Software Engineering (ICSE).

[11] Lukasczyk S., Fraser G. Pynguin: Automated unit test generation for python. Proceedings of the ACM/IEEE 44th International Conference on Software Engineering: Companion Proceedings.

[12] Mastropaolo A., Scalabrino S., Cooper N., Nader-Palacio D., Poshyvanyk D., Oliveto R., Bavota G. (2021). Studying the Usage of Text-To-Text Transfer Transformer to Support Code-Related Tasks. Proceedings of the 43rd IEEE/ACM International Conference on Software Engineering, ICSE 2021.

[13] Nie P., Banerjee R., Li J.J., Mooney R.J., Gligoric M. (2023). Learning Deep Semantics for Test Completion. Proceedings of the 45th IEEE/ACM International Conference on Software Engineering, ICSE 2023.

[14] Watson C., Tufano M., Moran K., Bavota G., Poshyvanyk D. (2020). On learning meaningful assert statements for unit test cases. Proceedings of the ICSE ’20: 42nd International Conference on Software Engineering.

[15] Chen Y., Hu Z., Zhi C., Han J., Deng S., Yin J. Chatunitest: A framework for llm-based test generation. Proceedings of the Companion Proceedings of the 32nd ACM International Conference on the Foundations of Software Engineering.

[16] Peng Y., Wang C., Wang W., Gao C., Lyu M.R. (2023). Generative Type Inference for Python. arXiv.

[17] ChatGPT. https://chat.openai.com/.

[18] CodeLlama. https://ollama.com/library/codellama.

[19] DeepSeek. https://www.deepseek.com/.

[20] Wang Y., Guo S., Tan C.W. (2025). From code generation to software testing: AI Copilot with context-based RAG. IEEE Softw..

[21] Ouyang L., Wu J., Jiang X., Almeida D., Wainwright C., Mishkin P., Zhang C., Agarwal S., Slama K., Ray A. (2022). Training language models to follow instructions with human feedback. Adv. Neural Inf. Process. Syst..

[22] Wong M.F., Tan C.W. (2024). Aligning crowd-sourced human feedback for reinforcement learning on code generation by large language models. IEEE Trans. Big Data.

